# Supplementary material for: Forced expression of MSR repeat transcripts above a threshold limit breaks heterochromatin organisation
Source: Nat Commun. 2025 Jul 11;16:6420. doi: 10.1038/s41467-025-61586-4 (PMC12254318; doi:10.1038/s41467-025-61586-4)
Supplement: Supplementary file 1 — Supplementary Information [file 41467_2025_61586_MOESM1_ESM.pdf]

SUPPLEMENTARY INFORMATION

Forced expression of MSR repeat transcripts above a threshold limit breaks heterochromatin organisation

Reagan W. Ching[1,*], Kalina M. Swist-Rosowska[1], Galina Erikson[1], Birgit Koschorz[1], Bettina Engist[1], and Thomas Jenuwein[1,*]

[1]Max Planck Institute of Immunobiology and Epigenetics (MPI-IE), Freiburg, Germany

*corresponding authors: ching@ie-freiburg.mpg.de and jenuwein@ie-freiburg.mpg.de

This file includes:

Supplementary Methods

Supplementary Figures 1-9

Supplementary Table 1

Supplementary References

SUPPLEMENTARY METHODS

Generation of dn57/Suv39h2-mCherry mESC

In a previous study[1], a mESC cell line was generated to express Suv39h2-EGFP in *Suv39h* double-null mESC (dn57). For this study, we used Suv39h2 tagged with an alternative fluorescent protein; i.e. the mCherry fluorescent protein. To generate dn57/Suv39h2-mCherry mESC cells, the pCAGGS-Suv39h2-mCherry-IRES-Puro plasmid (see Cloning section below) was linearized with PvuI (NEB) and 5 μg of the linearized plasmid was transfected into dn57 with Xfect (Clontech). 48 h after transfection, cells were selected with mESC medium containing 1 μg/ml puromycin for 7 days. FACS was performed to enrich for Suv39h2-mCherry expressing mESC cells, and the mESC cells were maintained as a mixed population.

Generation of dn57/Suv39h2-mCherry/T-Onc-EGFP mESC

To generate mESC cells that can inducibly express T-Onc-EGFP, dn57/Suv39h2-mCherry mESC cells were transfected with the plasmid expressing the PiggyBac transposase (SBI) together with the pPB-TRE-TALYM3B15-Onconase-EGFP plasmid (see Cloning section below) using Xfect (Clontech). 48 h after transfection, mESC were selected with complete mESC medium containing 100 μg/ml hygromycin. The same procedure was followed for the creation of the inducible T-EGFP control mESC, except the pPB-TRE-TALYM3B15-EGFP plasmid was transfected with the PiggyBac transposase plasmid. The mESC cells were maintained as a mixed population. T-EGFP and T-Onc-EGFP expression was confirmed by western blot and immunofluorescence microscopy using the GFP antibody.

Cell culture of mESC

mESC cells were cultured on 2% gelatin-coated dishes and maintained in high glucose DMEM medium (Sigma-Aldrich) containing 15% Serum Replacement (Thermo Fisher Scientific), 100 U/ml penicillin and 100 μg/μl streptomycin (Sigma- Aldrich), 2 mM L-glutamine (Sigma-Aldrich), 0.1 mM beta-mercaptoethanol, 1x non-essential amino acids (Sigma-Aldrich), 1 mM Na-pyruvate (Sigma-Aldrich), and 1 ml of homemade Leukemia Inhibitory Factor (LIF). For the culturing of dn57/Suv39h2-mCherry rescued mESC cells, mESC medium was supplemented with 1 μg/ml puromycin (Sigma-Aldrich). For the culturing of inducible dn57/Suv39h2-mCherry/T-EGFP and dn57/Suv39h2-mCherry/T-Onc-EGFP cells, mESC medium was supplemented with 1 μg/ml puromycin (Sigma-Aldrich) and 100 μg/ml hygromycin (Sigma-Aldrich).

Cloning of Suv39h2-mCherry

To generate the pCAGGS-Suv39h2-mCherry-IRES-Puro plasmid, the mCherry coding sequence was PCR-amplified from the pCI-Neo_K9-mCherry plasmid (Jenuwein Laboratory, #834) and ligated into the AgeI-NotI digested pCAGGS-Suv39h2-EGFP-IRES-Puro[1] to replace the EGFP coding sequence with the mCherry coding sequence. Plasmids were sequence verified.

Cloning of inducible T-Onc-EGFP

To generate the plasmid that contains the doxycycline inducible T-Onc-EGFP, the doxycycline inducible T-EGFP control plasmid was first created. The T-EGFP coding sequence was PCR-amplified from the pTALYM3B15 plasmid[2] (Addgene, #47878) and cloned into the NheI-PmeI digested pPB-TRE-dCas9-VPR (Addgene, #63800) by Gibson assembly to generate the pPB-TRE-TALYM3B15-EGFP plasmid. The Onconase-EGFP coding sequence was purchased as a synthetic gene from Integrated DNA Technologies (IDT), PCR-amplified, and cloned into the BamHI digested pPB-TRE-TALYM3B15-EGFP plasmid by Gibson assembly. Plasmids were sequence verified.

Western Blot analysis for inducible T-EGFP or T-Onc-EGFP

dn57/Suv39h2-mCherry/T-EGFP and dn57/Suv39h2-mCherry/T-Onc-EGFP mESC were induced with 0, 10, or 100 ng/ml doxycycline for 48 h. Cells were then trypsinised, harvested, and washed twice with PBS. To prepare protein lysates, cell pellets were lysed in RIPA buffer (Pierce) containing protease inhibitors (cOmplete EDTA-free protease inhibitors, Roche), and sonicated (30s ON, 30s OFF, 15 cycles) (Bioruptor, Diagenode). These samples were then processed for western blotting with antibodies against GFP (600-141-215, Rockland, 1:1000), and GAPDH (sc-32233, Santa Cruz, 1:1000).

RT-qPCR analysis for MSR expression in inducible T-EGFP or T-Onc-EGFP mESC

dn57/Suv39h2-mCherry/T-EGFP and dn57/Suv39h2-mCherry/T-Onc-EGFP mESC were induced with 10 ng/ml doxycycline for 48 h and then processed for RT-qPCR. Total RNA was extracted with TRI Reagent (Sigma-Aldrich, 93289). The remaining DNA was digested with TURBO DNase (ThermoFisher Scientific, AM2238), followed by clean-up with RNA Clean & Concentrator (Zymo Research, R1013). cDNA was created using Maxima reverse transcriptase (ThermoFisher Scientific, EP0741) using random hexamers following the manufacturers protocol. qPCR mixes contained diluted cDNA with 2X SYBR Select Master Mix (ThermoFisher Scientific, 4472920) and 200 nM of target-specific forward and reverse primers (See Supplementary Table 1) in a total volume of 10 μl. qPCR was performed using

a QuantStudio 6 Flex qPCR machine (Applied Biosystems). Cycle threshold (Ct) values were used to calculate normalised expression ($\Delta\Delta$Ct method).

Immunofluorescence microscopy of inducible T-EGFP or T-Onc-EGFP mESC

dn57/Suv39h2-mCherry/T-EGFP and dn57/Suv39h2-mCherry/T-Onc-EGFP mESC were induced with 10 ng/ml doxycycline for 48 h. mESC cells were harvested and immobilized on glass slides using a Cytospin (Thermo Scientific). After immobilization, slides were washed with PBS, fixed at room temperature with 4% PFA for 15 min, and permeabilized with 0.5% Triton X-100 for 5 min. Samples were then simultaneously stained using the antibodies GFP (600-141-215, Rockland, 1:1000) and mCherry (M11217, Invitrogen, 1:1000), and subsequently mounted with VECTASHIELD (Vector Laboratories, H-1200-10) containing DAPI. Images were acquired at 63x magnification with an LSM780 confocal microscope (Zeiss). Maximum intensity projections of the mESC were made with the ZEN Black software (Zeiss).

Time course of MSR-dCas9-effectors post-induction

MSR-dCas9-effectors MEF cells were induced with doxycycline (24 h for MSR-dCas9-Control and MSR-dCas9-Repressor, 6 h for MSR-dCas9-Activator). After induction, cells were washed with PBS and replaced with fresh media without doxycycline. Samples were collected at the following times: 1) Prior to induction (uninduced), 2) After induction (induced), and 3) 3, 6, 12 and 24 h post-induction (3, 6, 12, 24, respectively). The samples at the various time points were then processed for western blot analysis and total RNA purification for RT-qPCR analysis and RNA sequencing (see Methods).

RNase A treatment

Microscope slides (Superfrost, Epredia) were coated with a solution of 0.7% low-melting-point agarose prepared in water and left to dry completely prior to cell harvesting. dCas9-MSR-Activator MEF cells were harvested by trypsinisation, washed and resuspended in PBS. This cell suspension was then mixed with an equal volume of 1.4% low melting point agarose prepared in PBS and kept at 37°C. 50 µl of the cell suspension was spotted onto the agarose-coated slides and covered with a 22x22 mm coverslip. The slides were then placed on a 37°C thermoblock for 3 min to allow the cells to sediment. The slides were then placed onto an ice-cold metal block for 3 min to solidify the agarose. Coverslips were gently removed and the slides were placed in a dish containing fresh culture medium with or without 10 ng/ml doxycycline for 3 or 4 h at 37°C in a tissue culture incubator. After the incubation, the slides were briefly washed in PBS and then cells were permeabilized with 0.5% Triton X-100 in PBS for 5 min. The slides were then washed 3x5 min with PBS and incubated for 1 h at 37°C with

20U of RNase A in digestion buffer (20 mM HEPES pH 7.5, 0.1 mM $CaCl_2$, 3 mM $MgCl_2$, 100 mM KCl). After RNA digestion, slides were washed 3x 5 min with PBS and then fixed with 4% PFA in PBS for 15 min. Slides were then stained with antibodies against NPM1 (Abcam, AB10530, 1:1000) and HP1a (Abcam, AB109028, 1:1000) and mounted with VECTASHIELD containing DAPI (Vector Laboratories, H-1200-10). Cells were imaged using an LSM880 confocal microscope (Zeiss) at 63x magnification. Maximum intensity projections were created with using the ZEN Black software (Zeiss). Analysis of the images were performed using Fiji (2.9.0).

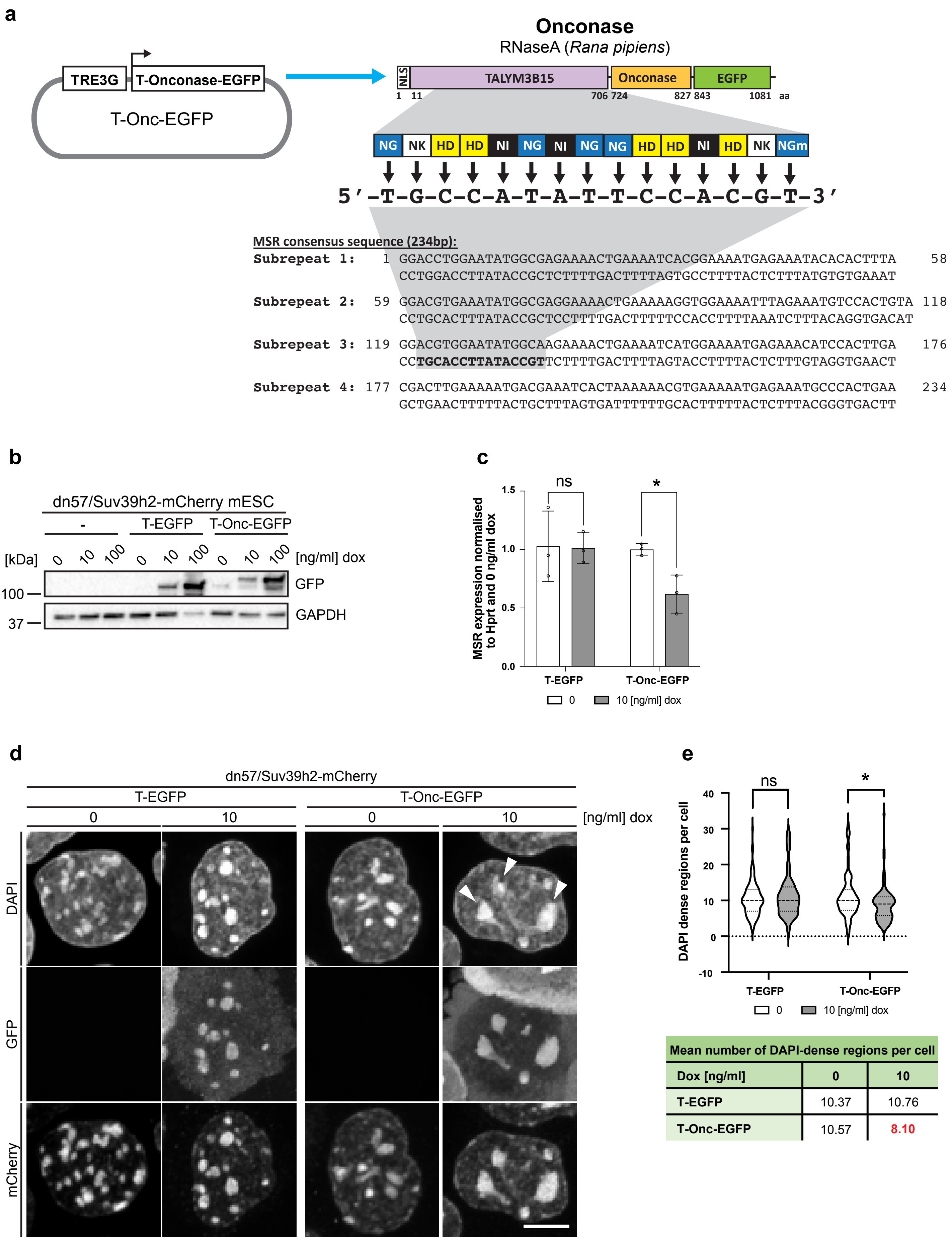
a
TRE3G
T-Onconase-EGFP
T-Onc-EGFP
Onconase
RNaseA (Rana pipiens)
NLS
TALYM3B15
Onconase
EGFP
1 11 706 724 827 843 1081 aa
NG NK HD HD NI NG NI NG NG HD HD NI HD NK NGm
5′-T-G-C-C-A-T-A-T-T-C-C-A-C-G-T-3′
MSR consensus sequence (234bp):
Subrepeat 1: 1 GGACCTGGAATATGGCGAGAAAACTGAAAATCACGGAAAATGAGAAATACACACTTTA 58
CCTGGACCTTATACCGCTCTTTTGACTTTTAGTGCCTTTTACTCTTTATGTGTGAAAT
Subrepeat 2: 59 GGACGTGAAATATGGCGAGGAAAACTGAAAAAGGTGGAAAATTTAGAAATGTCCACTGTA 118
CCTGCACTTTATACCGCTCCTTTTGACTTTTTCCACCTTTTAAATCTTTACAGGTGACAT
Subrepeat 3: 119 GGACGTGGAATATGGCAAGAAAACTGAAAATCATGGAAAATGAGAAACATCCACTTGA 176
CCTGCACCTTATACCGTTCTTTTGACTTTTAGTACCTTTTACTCTTTGTAGGTGAACT
Subrepeat 4: 177 CGACTTGAAAAATGACGAAATCACTAAAAAACGTGAAAAATGAGAAATGCCCACTGAA 234
GCTGAACTTTTTACTGCTTTAGTGATTTTTTGCACTTTTTACTCTTTACGGGTGACTT
b
dn57/Suv39h2-mCherry mESC
-
T-EGFP
T-Onc-EGFP
[kDa]
0 10 100 0 10 100 0 10 100
[ng/ml] dox
100
37
GFP
GAPDH
c
MSR expression normalised to Hprt and 0 ng/ml dox
1.5
1.0
0.5
0.0
ns
*
T-EGFP
T-Onc-EGFP
0
10 [ng/ml] dox
d
dn57/Suv39h2-mCherry
T-EGFP
T-Onc-EGFP
0
10
0
10
[ng/ml] dox
DAPI
GFP
mCherry
e
DAPI dense regions per cell
40
30
20
10
0
-10
ns
*
T-EGFP
T-Onc-EGFP
0
10 [ng/ml] dox
Mean number of DAPI-dense regions per cell
Dox [ng/ml] | 0 | 10
T-EGFP | 10.37 | 10.76
T-Onc-EGFP | 10.57 | 8.10

Supplementary Figure 1. MSR targeting of T-Onconase-EGFP (T-Onc-EGFP). a Schematic representation of the T-Onc-EGFP targeting construct. A Tet-ON system was used to induce the expression of full-length amphibian (*Rana pipiens*) RNase A (Onconase) fused to NLS-TALYM3B15 (Miyanari et al., 2013) and EGFP. TALYM3B15 is a DNA binding domain composed of 15 units that recognise 15 nt in the reverse strand of MSR subrepeat 3 (indicated in bold). **b** Western blot for the detection of T-EGFP and T-Onc-EGFP in dn57/Suv39h2-mCherry mESC. T-EGFP and T-Onc-EGFP plasmids were stably transfected into *Suv39h* double-null mESC (dn57) rescued with Suv39h2-mCherry (see Methods). T-EGFP and T-Onc-EGFP were induced with increasing concentrations of doxycycline (0, 10, and 100 ng/ml) and their expression was probed with an α-GFP antibody. GAPDH expression is shown as a loading control. **c** RT-qPCR analysis for MSR transcripts 48 h after induction (dox 10 ng/ml) of T-EGFP or T-Onc-EGFP in dn57/Suv39h2-mCherry mESC. MSR expression is normalised to *Hprt* and relative to each uninduced (dox 0 ng/ml) sample (mean±SD). The asterisk indicates a statistically significant difference (*, $p \leq 0.033$, two-way ANOVA, Fisher's LSD test). n=3 independent experiments. **d** Double immunofluorescence for the localisation of induced T-EGFP and T-Onc-EGFP in dn57/Suv39h2-mCherry mESC. T-EGFP and T-Onc-EGFP were detected with an α-GFP antibody, Suv39h2-mCherry with an α-mCherry antibody and cells were counterstained with DAPI. The white arrowheads indicate aggregated DAPI-dense regions. Scale bar is 5 μm. **e** Violin plots to quantify the number of DAPI-dense regions per cell for the conditions imaged in (d) (DAPI counterstaining). The asterisk indicates a statistically significant difference (*, $p \leq 0.033$, two-way ANOVA, Tukey's test). The table specifies the mean number of DAPI-dense regions per cell in uninduced and induced T-EGFP-dn57/Suv39h2-mCherry and T-Onc-EGFP-dn57/Suv39h2-mCherry mESC. For each sample, n>70 cells were analysed.

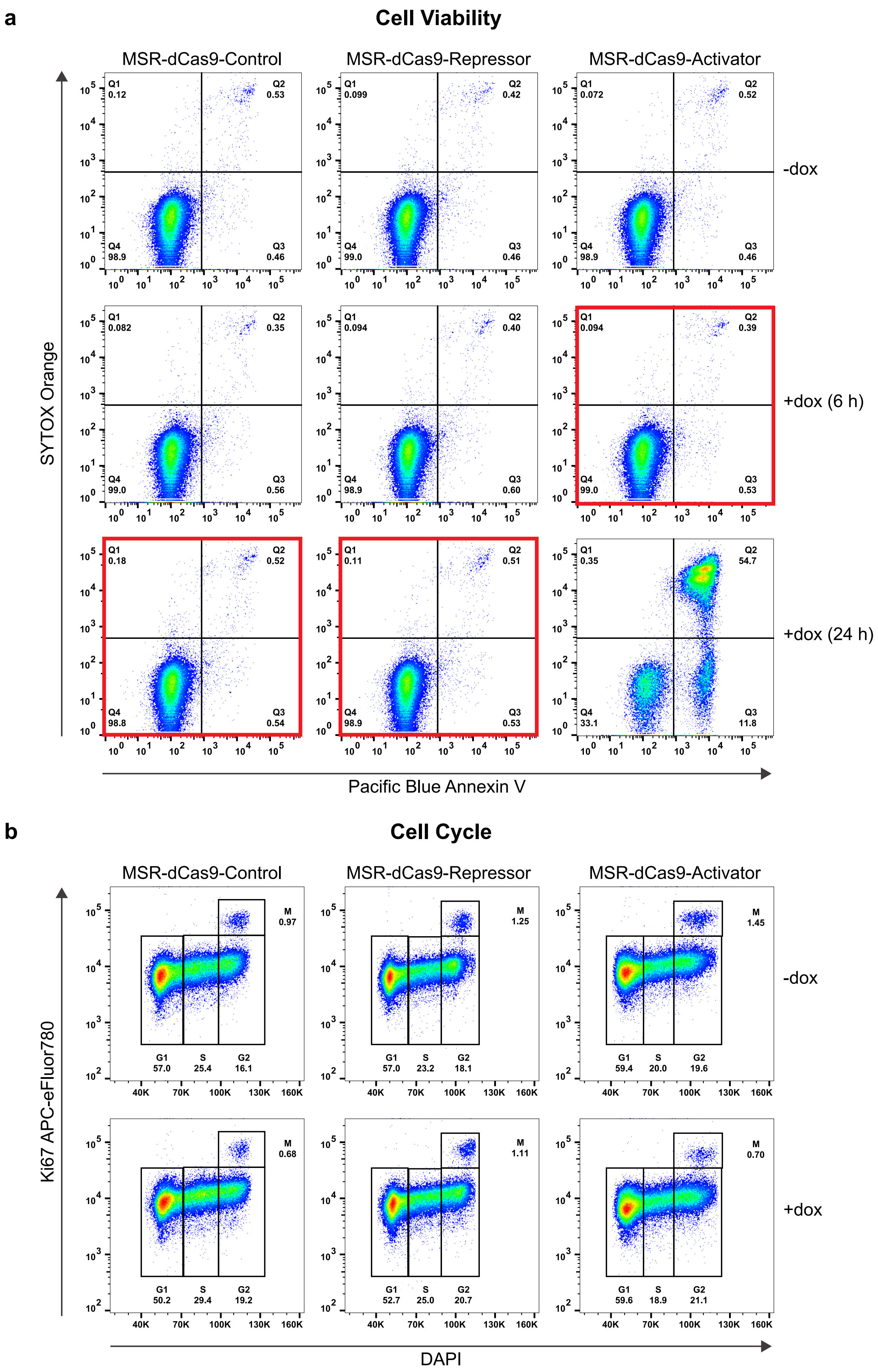
a
Cell Viability
MSR-dCas9-Control
MSR-dCas9-Repressor
MSR-dCas9-Activator
-dox
+dox (6 h)
+dox (24 h)
SYTOX Orange
Pacific Blue Annexin V
Q1 0.12 Q2 0.53 Q4 98.9 Q3 0.46
Q1 0.099 Q2 0.42 Q4 99.0 Q3 0.46
Q1 0.072 Q2 0.52 Q4 98.9 Q3 0.46
Q1 0.082 Q2 0.35 Q4 99.0 Q3 0.56
Q1 0.094 Q2 0.40 Q4 98.9 Q3 0.60
Q1 0.094 Q2 0.39 Q4 99.0 Q3 0.53
Q1 0.18 Q2 0.52 Q4 98.8 Q3 0.54
Q1 0.11 Q2 0.51 Q4 98.9 Q3 0.53
Q1 0.35 Q2 54.7 Q4 33.1 Q3 11.8
b
Cell Cycle
MSR-dCas9-Control
MSR-dCas9-Repressor
MSR-dCas9-Activator
-dox
+dox
Ki67 APC-eFluor780
DAPI
40K 70K 100K 130K 160K
M 0.97 G1 57.0 S 25.4 G2 16.1
M 1.25 G1 57.0 S 23.2 G2 18.1
M 1.45 G1 59.4 S 20.0 G2 19.6
M 0.68 G1 50.2 S 29.4 G2 19.2
M 1.11 G1 52.7 S 25.0 G2 20.7
M 0.70 G1 59.6 S 18.9 G2 21.1

Supplementary Figure 2. Cell viability and cell cycle analysis of MSR-dCas9-effector component MEF cells. a Flow cytometry analysis for cell viability of MSR-dCas9-Control, MSR-dCas9-Repressor and MSR-dCas9-Activator MEF cells uninduced or induced with doxycycline (dox) for 6 h or 24 h. Cells were stained with SYTOX Orange for viability and then stained for Annexin V for apoptosis. Induction conditions that maintain cell viability are 24 h for MSR-dCas9-Control and MSR-dCas9-Repressor, and 6 h for MSR-dCas9-Activator (marked by red boxes). **b** Flow cytometry analysis for cell cycle stages of MSR-dCas9-Control, MSR-dCas9-Repressor and MSR-dCas9-Activator MEF cells uninduced or induced with doxycycline (dox) and stained for Ki67 and DAPI. MSR-dCas9-Control and MSR-dCas9-Repressor were induced for 24 h, and MSR-dCas9-Activator was induced for 6 h.

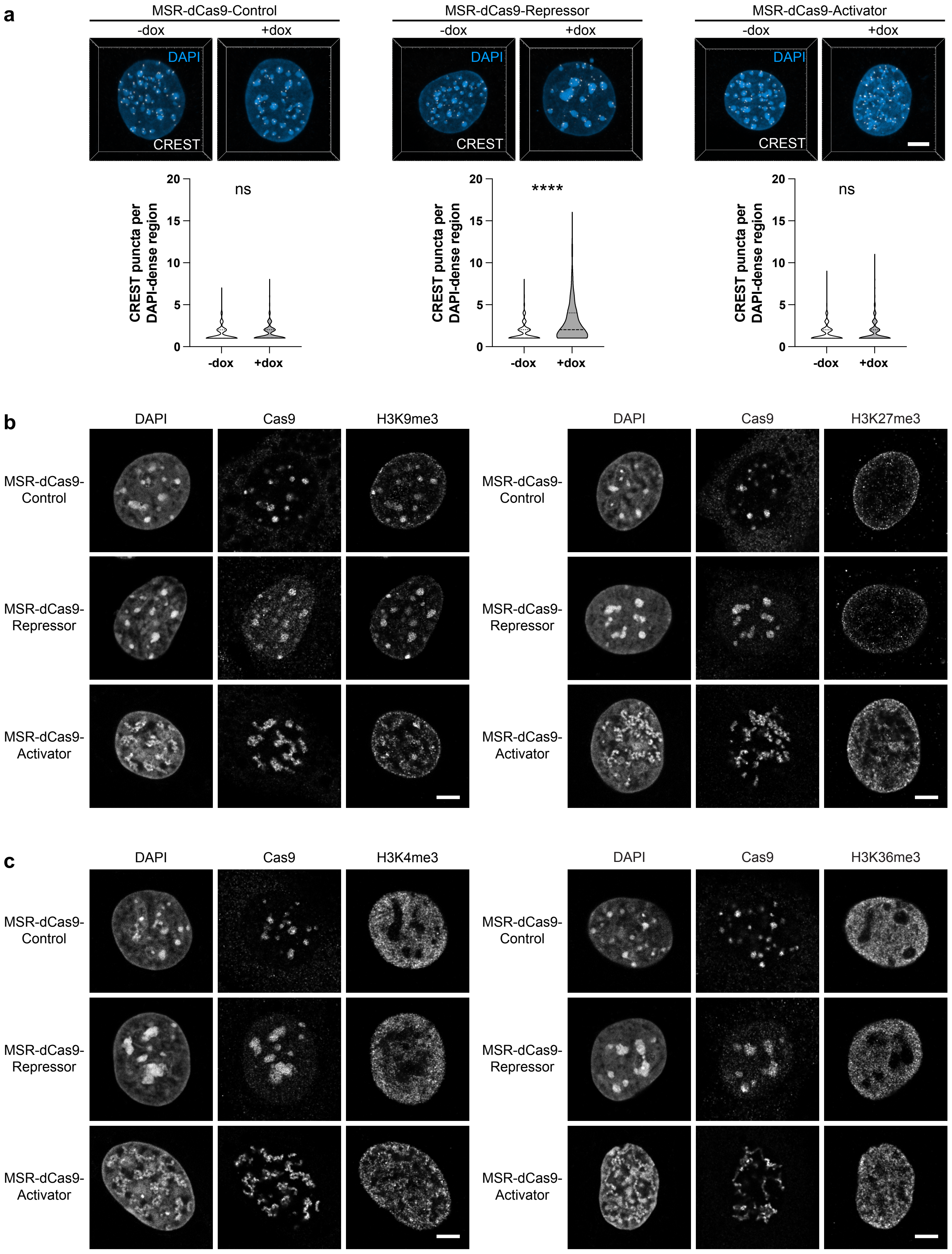
a
MSR-dCas9-Control
MSR-dCas9-Repressor
MSR-dCas9-Activator
-dox
+dox
DAPI
CREST
CREST puncta per DAPI-dense region
0
5
10
15
20
ns
****
b
DAPI
Cas9
H3K9me3
H3K27me3
MSR-dCas9-Control
MSR-dCas9-Repressor
MSR-dCas9-Activator
c
DAPI
Cas9
H3K4me3
H3K36me3
MSR-dCas9-Control
MSR-dCas9-Repressor
MSR-dCas9-Activator

Supplementary Figure 3. Double immunofluorescence for CREST puncta and H3 methylation marks in MSR-dCas9-effector MEF cells. a Immunofluorescence microscopy of MEF cells uninduced or induced for the expression of MSR-dCas9-Control, MSR-dCas9-Repressor and MSR-dCas9-Activator. Cells were immunostained using CREST antiserum (white puncta) and counterstained with DAPI (blue). Shown are representative images (merged z-stacks) of CREST with DAPI. Scale bar is 5 μm. The number of CREST puncta per DAPI-dense region were counted and are shown as violin plots below. For each sample, n≥390 DAPI-dense regions were quantified from n=3 independent experiments. The asterisks indicate a statistically significant difference (****, p<0.0001, ns, not significant, two-sided Mann-Whitney test). **b** Double immunofluorescence in inducible MSR-dCas9-Control, MSR-dCas9-Repressor and MSR-dCas9-Activator MEF cells for the localisation of MSR-dCas9-effector components and H3K9me3 (left panel) or for MSR-dCas9-effector components and H3K27me3 (right panel). Nuclei were counterstained with DAPI. For each sample n≥20 cells were analysed. Scale bar is 5 μm. **c** Double immunofluorescence in inducible MSR-dCas9-Control, MSR-dCas9-Repressor and MSR-dCas9-Activator MEF cells for localisation of MSR-dCas9-effector components and H3K4me3 (left panel) or for MSR-dCas9-effector components and H3K36me3 (right panel). Nuclei were counterstained with DAPI. For each sample n≥20 cells were analysed. Scale bar is 5 μm.

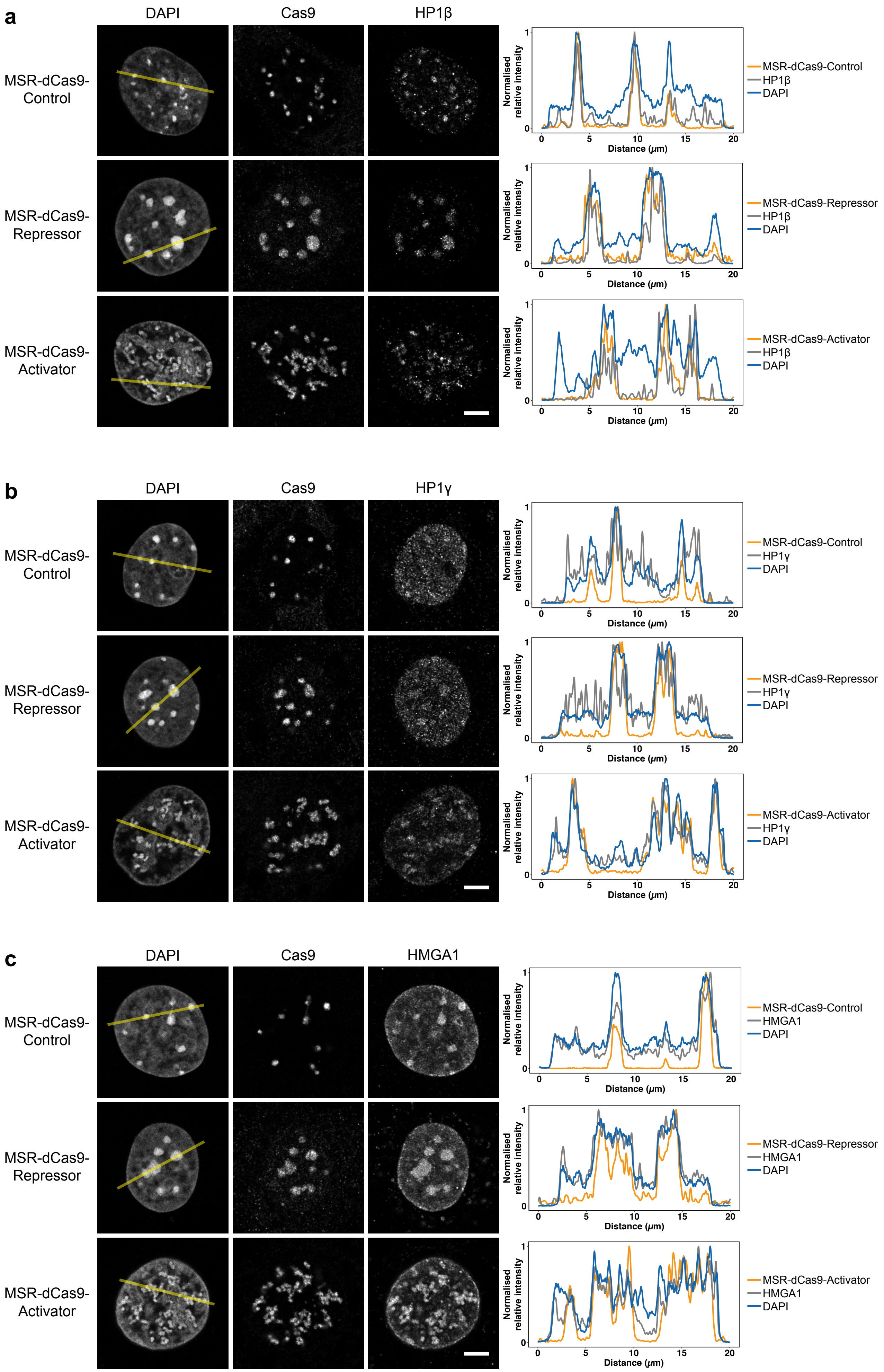

a
DAPI
Cas9
HP1β
MSR-dCas9-Control
MSR-dCas9-Repressor
MSR-dCas9-Activator
Normalised relative intensity
Distance (μm)
MSR-dCas9-Control
HP1β
DAPI
MSR-dCas9-Repressor
HP1β
DAPI
MSR-dCas9-Activator
HP1β
DAPI
b
DAPI
Cas9
HP1γ
MSR-dCas9-Control
MSR-dCas9-Repressor
MSR-dCas9-Activator
Normalised relative intensity
Distance (μm)
MSR-dCas9-Control
HP1γ
DAPI
MSR-dCas9-Repressor
HP1γ
DAPI
MSR-dCas9-Activator
HP1γ
DAPI
c
DAPI
Cas9
HMGA1
MSR-dCas9-Control
MSR-dCas9-Repressor
MSR-dCas9-Activator
Normalised relative intensity
Distance (μm)
MSR-dCas9-Control
HMGA1
DAPI
MSR-dCas9-Repressor
HMGA1
DAPI
MSR-dCas9-Activator
HMGA1
DAPI

Supplementary Figure 4. Double immunofluorescence for core heterochromatin components in MSR-dCas9-effector MEF cells. a-c Immunofluorescence microscopy was performed on MEF cells uninduced or induced for the expression of MSR-dCas9-Control, MSR-dCas9-Repressor and MSR-dCas9-Activator MEF cells. Cells were double-stained for Cas9 and for the heterochromatic proteins HP1β (**a**), HP1γ (**b**) or HMGA1 (**c**). Nuclei were counterstained with DAPI. Scale bar is 5 μm. For each sample n≥21 cells were analysed. Shown on the right are linescans of the representative images on the left. Linescans were used to visualise the overlap of DAPI (blue), Cas9 (orange), and HP1β (**a**), HP1γ (**b**) or HMGA1 (**c**) (gray) signals.

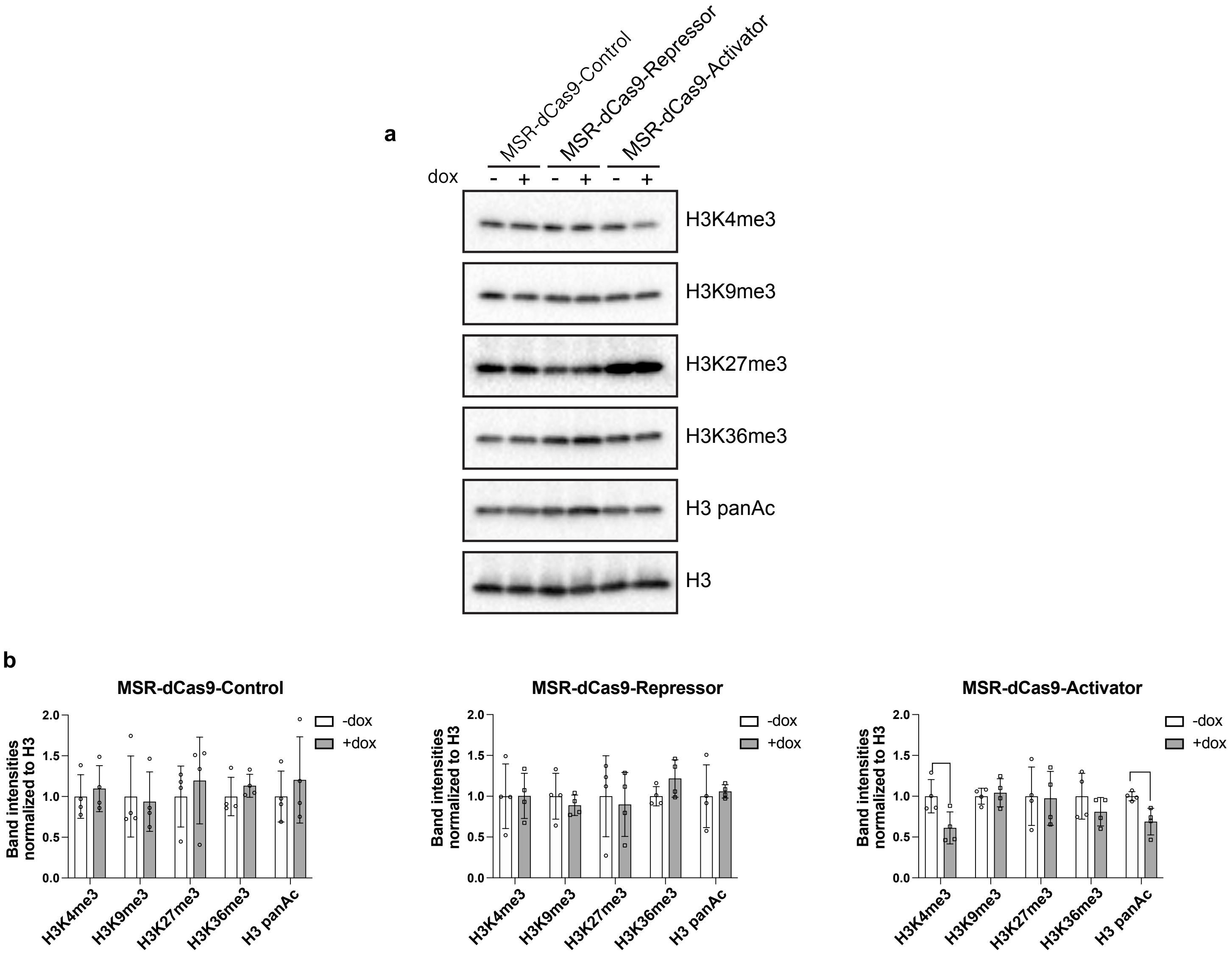

Supplementary Figure 5. Bulk levels of H3K4me3, H3K9me3, H3K27me3, H3K36me3, and H3 panAc in the MSR-dCas9-effector MEF cells. a Western blot for total histone H3 and the H3 modifications H3K4me3, H3K9me3, H3K27me3, H3K36me3 and H3 pan-acetyl (H3 panAc). Whole cell extracts were derived from MSR-dCas9-Control, MSR-dCas9-Repressor and MSR-dCas9-Activator MEF cells uninduced or induced with doxycycline (dox). Histone H3 staining is shown as a loading control. **b** Bar graphs show the band intensity quantification from the Western blot in (**a**). Band intensities were normalised to the band intensity of unmodified H3 (mean±SD). Asterisks indicate statistically significant differences (*, $p \leq 0.05$, two-sided multiple t-tests). n=4 independent experiments.

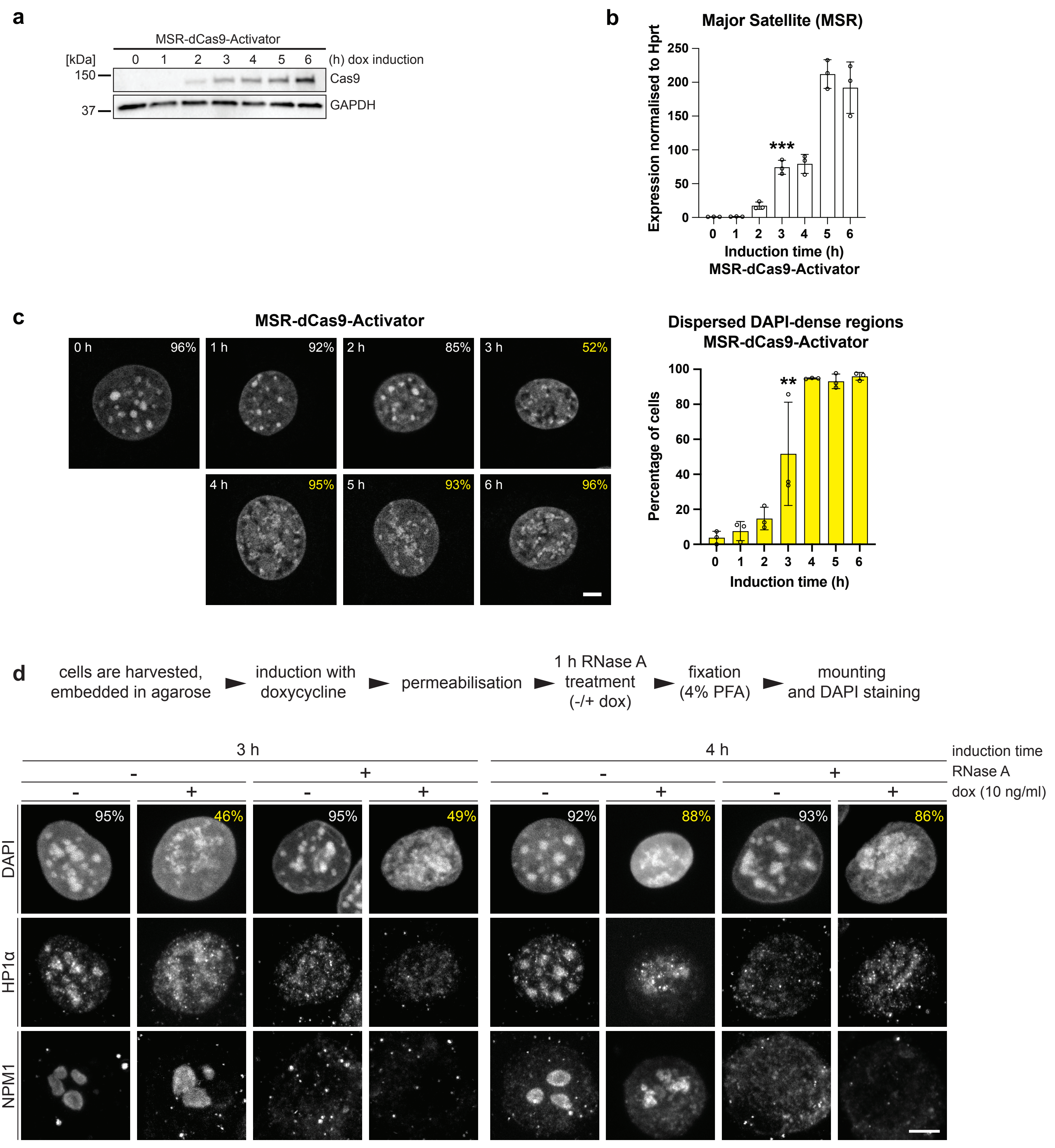
a
MSR-dCas9-Activator
[kDa]
0 1 2 3 4 5 6 (h) dox induction
150
Cas9
37
GAPDH
b
Major Satellite (MSR)
Expression normalised to Hprt
250
200
150
100
50
0
***
0 1 2 3 4 5 6
Induction time (h)
MSR-dCas9-Activator
c
MSR-dCas9-Activator
0 h 96%
1 h 92%
2 h 85%
3 h 52%
4 h 95%
5 h 93%
6 h 96%
Dispersed DAPI-dense regions
MSR-dCas9-Activator
Percentage of cells
100
80
60
40
20
0
**
0 1 2 3 4 5 6
Induction time (h)
d
cells are harvested, embedded in agarose
induction with doxycycline
permeabilisation
1 h RNase A treatment (-/+ dox)
fixation (4% PFA)
mounting and DAPI staining
3 h
4 h
induction time
RNase A
dox (10 ng/ml)
DAPI
HP1α
NPM1
95%
46%
95%
49%
92%
88%
93%
86%

Supplementary Figure 6. Time course for induction of MSR-dCas9-Activator and RNase A digestion of permeabilised MSR-dCas9-Activator MEF cells. a Western blot analysis of the expression of MSR-dCas9-Activator induced for 0, 1, 2, 3, 4, 5, 6 h with doxycycline (10 ng/ml). GAPDH staining is shown as a loading control. **b** RT-qPCR analysis for MSR transcripts at the increasing induction times of MSR-dCas9-Activator. Values were normalised to *Hprt* and are relative to the MSR-dCas9-Activator (-dox, 0 h induction point) (mean±SD). Asterisks indicate a statistically significant difference (***, $p \leq 0.0010$, one-way ANOVA, Dunnett's test) for MSR transcript deregulation at the 3 h time point where around 50% of the cells display dispersed DAPI-dense regions (see (c), below). n=3 independent experiments. **c** Confocal imaging of DAPI-dense regions (DAPI counterstaining) in MSR-dCas9-Activator MEF cells with increasing induction time (mean±SD). (**, $p \leq 0.0010$, one-way ANOVA, Dunnett's test). n=3 independent experiments. Scale bar is 5 μm. The percentages reflect the fraction of cells with either undispersed (white) or dispersed (yellow) DAPI-dense regions. Quantification of the imaging data is shown in the bar graph to the right. For each time point, n≥150 cells were analysed from three independent experiments. **d** A flow diagram for the processing of MSR-dCas9-Activator MEF cells is shown on top. Cells were embedded in agarose, induced, permeabilised and then incubated with RNase A. Processed cells were double-labelled for HP1α and nucleophosmin (NPM1) and counterstained with DAPI. Scale bar is 5 μm. The percentages reflect the fraction of cells with either undispersed (white) or dispersed (yellow) DAPI-dense regions. For each sample n≥50 cells were analysed.

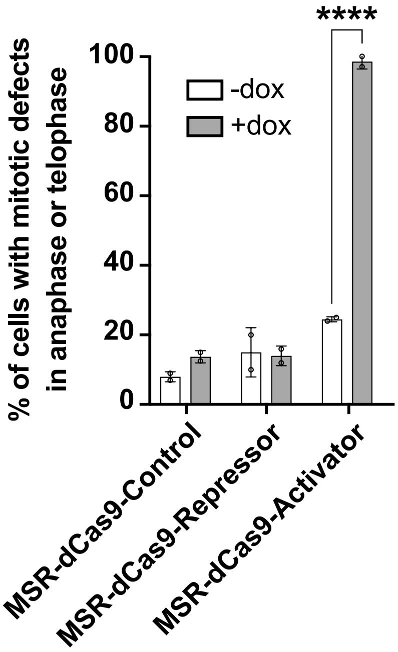

Supplementary Figure 7. Quantification of mitotic defects in MSR-dCas9-effector MEF cells. The bar graph shows the quantification of mitotic defects in anaphase and telophase of uninduced or induced MSR-dCas9-effector MEF cells (mean±SD), as they were analysed by confocal imaging (see Figure 6c). For each sample, n≥20 cells in anaphase and telophase were quantified from two independent experiments. The asterisks indicate a statistically significant difference (****, $p<0.0001$, two-way ANOVA, Tukey's test).

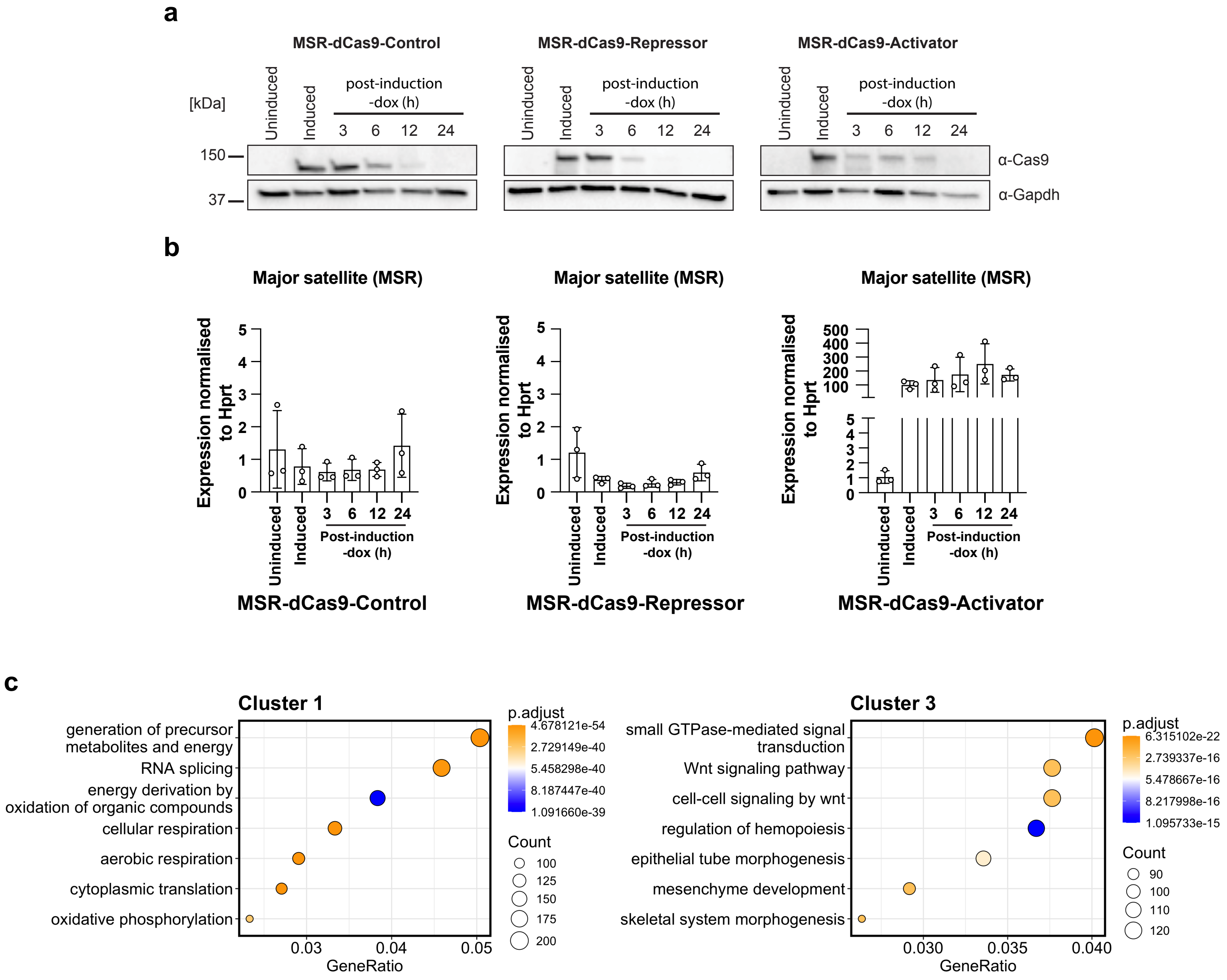

Supplementary Figure 8. Stability of MSR-dCas9-effector components post-induction. **a** Western blot analysis to examine the stability of MSR-dCas9-Control, MSR-dCas9-Activator and MSR-dCas9-Repressor. Cells were induced with doxycycline (dox) and protein expression was analysed 3, 6, 12 and 24 h after doxycycline removal. GAPDH staining is shown as a loading control. **b** RT-qPCR analysis for MSR transcripts at post-induction time points in MSR-dCas9-Control, MSR-dCas9-Repressor and MSR-dCas9-Activator MEF cells. For each histogram, values are normalised to *Hprt* and are relative to the uninduced sample (mean±SD). n=3 independent experiments. **c** Gene ontology (GO) analysis of clusters 1 and 3 from the heatmap shown in Figure 7b. Dot plots display the top 7 GO terms for each cluster. n=3 independent experiments.

a

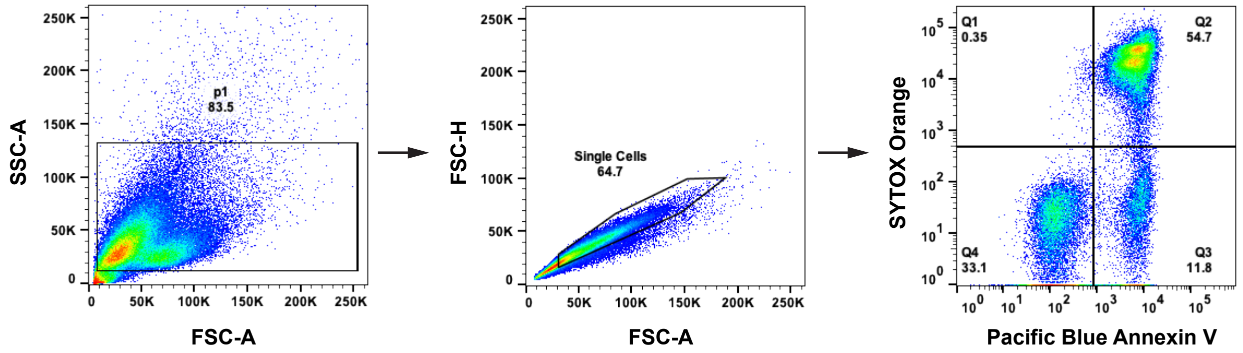

b

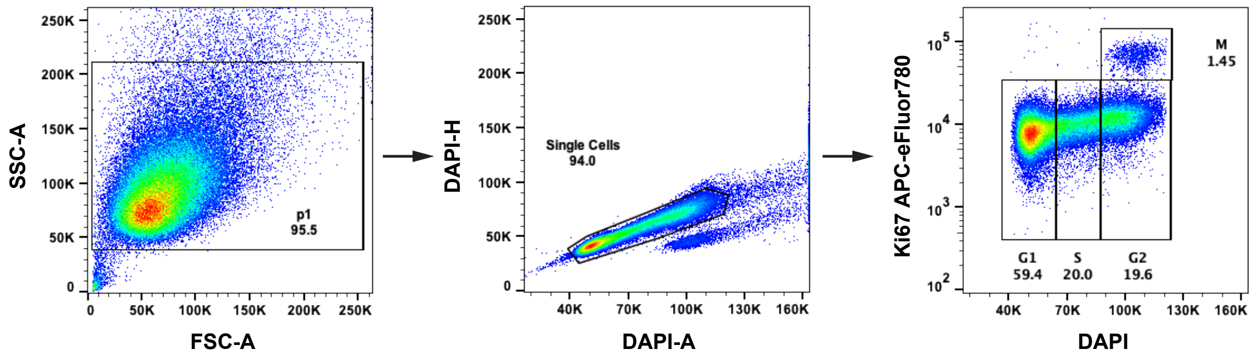

Supplementary Figure 9. Gating strategy for cell viability and cell cycle flow cytometry analysis. a MSR-dCas9-effector cells flow cytometry gating strategy used to calculate the proportion of apoptotic and dead cells. Cell viability was determined using SYTOX Orange and apoptosis with Pacific Blue Annexin V antibody. **b** MSR-dCas9-effector cells flow cytometry gating strategy used to indicate the different phases of the cell cycle. DNA content was determined by DAPI and the mitotic fraction with Ki67 APC-eFluor780 antibody.

Supplementary Table 1: List of oligonucleotides sequences used in the study

Oligo name	Type	Sequence
gMSR3 F	Cloning	CTGATGACCGGTGAAATGTCCACTGTAGGACGG
gRNA R	Cloning	CATCAGGAATTCAAAAAAAGCACCGACTCGGT
gMSR3	Cloning	CTGATGACCGGTGAAATGTCCACTGTAGGACGGGTTTTAGAGCTAGAAATAGCAAGTTAAAATAAGGCTAGTCCGTTATCAACTTGAAAAAGTGGCACCGAGTCGGTGCTTTTTTTGAATTCCTGATG
hU6 F	Cloning	GAGGGCCTATTTCCCATGATT
SpeI U6 gRNA R	Cloning	GATCTTACTAGTGAGGGCCTATTTCCCATGATT
SpeI U6 gRNA R	Cloning	AGCAATACTAGTCTCTAGAGCCATTTGTCTGCA
AgeI_mCherry_for	Cloning	CGCGCGACCGGTCGCCACCATGGTGAGCAAGGGCGAGGAG
mCherry_NotI_rev	Cloning	GCGCGCGCGGCCGCTTACAGCTCGTCCATGCCGCCGGT
PB-T_for	Cloning	ACCCTCGTAAAGGTCTAGAGATGAGATCTCCTAAGAAAAAG
T-PB_rev	Cloning	TCAGTTAGCCTCCCCCGTTTTTAGCATTTAGGTGACAC
GA onconase_for	Cloning	CACGTCCCATCGCGTTGCGGGATCCGGTGGAGGAGGTTC
GA onconase_rev	Cloning	GATCCACTTAGATTTAGCTGCGATTTCGCAGATCTGCTATG
MSR	qPCR	Fw: TGGAATATGGCGAGAAAACTG<br>Rv: AGGTCCTTCAGTGGGCATTT
L1Md_A	qPCR	Fw: ACTGCGGTACATAGGGAAGC<br>Rv: TGTGATCCACTCACCAGAGG
Minor satellite	qPCR	Fw: TTGGAAACGGGATTTGTAGA<br>Rv: CGGTTTCCAACATATGTGTTTT
Hprt	qPCR	Fw: AGTGATAGATCCATTCCTATGACTGTAG<br>Rv: GTTAAAGTTGAGAGATCATCTCCACC
MSR (Sense mix) *	RNA FISH	1: Biot-aAtAtGgCaAgAaAaCtGaAaAT<br>2: Biot-aAtGaGaAaCaTcCaCtTgAcGaCT<br>3: Biot-tGaAaAaTgAgAaAtGcAcAcTG
MSR (Antisense mix) *	RNA FISH	1: Biot-ATtTtCaGtTtTcCaTaTt<br>2: Biot-AGtCgTcAaGtGgAtGtTtCtCaTt<br>3: Biot-cAgTgTgCaTtTcTcAtTtTtCa
MSR *	DNA FISH	1: Biot-aTtTaGaAaTgTcCaCtGtAgGaC<br>2: Biot-aAtAtGgCaAgAaAaCtGaAaAT<br>3: Biot-aAtGaGaAaCaTcCaCtTgAcGaCT<br>4: Biot-tGaAaAaTgAgAaAtGcAcAcTG

* For FISH probes, lower case indicates Locked Nucleic Acid (LNA)

REFERENCES

1. Velazquez Camacho O., et al. Major satellite repeat RNA stabilize heterochromatin retention of Suv39h enzymes by RNA-nucleosome association and RNA:DNA hybrid formation. *Elife* **6**, e25293 (2017).

2. Miyanari Y., Ziegler-Birling C. & Torres-Padilla M. E. Live visualization of chromatin dynamics with fluorescent TALEs. *Nat Struct Mol Biol* **20**, 1321-1324 (2013).
