# Supplementary material for: Forced expression of MSR repeat transcripts above a threshold limit breaks heterochromatin organisation
Source: Nat Commun. 2025 Jul 11;16:6420. doi: 10.1038/s41467-025-61586-4 (PMC12254318; doi:10.1038/s41467-025-61586-4)
Supplement: Supplementary file 3 — Reporting Summary [file 41467_2025_61586_MOESM3_ESM.pdf]

nature portfolio

Corresponding author(s): Reagan Ching and Thomas Jenuwein

Last updated by author(s): Jun 5, 2025

# Reporting Summary

Nature Portfolio wishes to improve the reproducibility of the work that we publish. This form provides structure for consistency and transparency in reporting. For further information on Nature Portfolio policies, see our Editorial Policies and the Editorial Policy Checklist.

## Statistics

For all statistical analyses, confirm that the following items are present in the figure legend, table legend, main text, or Methods section.

| n/a | Confirmed | |
|---|---|---|
| ☐ | ☒ | The exact sample size (*n*) for each experimental group/condition, given as a discrete number and unit of measurement |
| ☐ | ☒ | A statement on whether measurements were taken from distinct samples or whether the same sample was measured repeatedly |
| ☐ | ☒ | The statistical test(s) used AND whether they are one- or two-sided<br>*Only common tests should be described solely by name; describe more complex techniques in the Methods section.* |
| ☒ | ☐ | A description of all covariates tested |
| ☒ | ☐ | A description of any assumptions or corrections, such as tests of normality and adjustment for multiple comparisons |
| ☐ | ☒ | A full description of the statistical parameters including central tendency (e.g. means) or other basic estimates (e.g. regression coefficient) AND variation (e.g. standard deviation) or associated estimates of uncertainty (e.g. confidence intervals) |
| ☐ | ☒ | For null hypothesis testing, the test statistic (e.g. *F*, *t*, *r*) with confidence intervals, effect sizes, degrees of freedom and *P* value noted<br>*Give P values as exact values whenever suitable.* |
| ☒ | ☐ | For Bayesian analysis, information on the choice of priors and Markov chain Monte Carlo settings |
| ☒ | ☐ | For hierarchical and complex designs, identification of the appropriate level for tests and full reporting of outcomes |
| ☒ | ☐ | Estimates of effect sizes (e.g. Cohen's *d*, Pearson's *r*), indicating how they were calculated |

*Our web collection on statistics for biologists contains articles on many of the points above.*

## Software and code

Policy information about availability of computer code

| | |
|---|---|
| Data collection | ZEN Blue 3.10.0005 (Zeiss), ZEN black 2012 SP5 (Zeiss). Image Lab 6.0.1 (Bio-Rad), QuantStudio Real-Time PCR software 1.1 (Applied Biosystems), FACSDiva 8.0.3 (BD), FIJI 2.9.0 (ImageJ), Axygen Gel Documentation system (Axygen) 1.1.1.0512 |
| Data analysis | FlowJo 10.8.1 (BD), FIJI (ImageJ) or Imaris 9.7.0 (Oxford instruments), Prism 10 (GraphPad), Image Lab 6.0.1 (Bio-Rad), FIJI 2.9.0 (ImageJ), Excel 16.83 (Microsoft), R packages used for RNA-seq analysis: DESeq2 1.34.0, cutadapt 1.8.1, TEtranscripts 2.0.3. |

For manuscripts utilizing custom algorithms or software that are central to the research but not yet described in published literature, software must be made available to editors and reviewers. We strongly encourage code deposition in a community repository (e.g. GitHub). See the Nature Portfolio guidelines for submitting code & software for further information.

## Data

Policy information about availability of data

All manuscripts must include a data availability statement. This statement should provide the following information, where applicable:

- Accession codes, unique identifiers, or web links for publicly available datasets
- A description of any restrictions on data availability
- For clinical datasets or third party data, please ensure that the statement adheres to our policy

The RNA-seq datasets generated in this study have been deposited in the GEO repository under the accession code GSE287837 (https://www.ncbi.nlm.nih.gov/geo/query/acc.cgi?acc=GSE287837). The data and any unique materials are available from the corresponding author upon reasonable request. All oligonucleotides used in this study are provided in Supplementary Table 1. Source data are provided with this paper.

## Research involving human participants, their data, or biological material

Policy information about studies with human participants or human data. See also policy information about sex, gender (identity/presentation), and sexual orientation and race, ethnicity and racism.

| | |
|---|---|
| Reporting on sex and gender | n/a |
| Reporting on race, ethnicity, or other socially relevant groupings | n/a |
| Population characteristics | n/a |
| Recruitment | n/a |
| Ethics oversight | n/a |

Note that full information on the approval of the study protocol must also be provided in the manuscript.

# Field-specific reporting

Please select the one below that is the best fit for your research. If you are not sure, read the appropriate sections before making your selection.

- [x] Life sciences
- [ ] Behavioural & social sciences
- [ ] Ecological, evolutionary & environmental sciences

For a reference copy of the document with all sections, see nature.com/documents/nr-reporting-summary-flat.pdf

# Life sciences study design

All studies must disclose on these points even when the disclosure is negative.

| | |
|---|---|
| Sample size | No statistical method was used to determine sample size. The standard of three biological replicates were chosen for western blot, qPCR, and RNA-seq analysis. Samples sizes for immunofluorescence imaging analysis are indicated in the appropriate figure legends. |
| Data exclusions | Data was not shown concerning the MSR RNA transcript analysis of mESCs expressing Cas13d because no affect on MSR RNA levels was observed. Data was also not shown for the experiment where RNase enzymes were targeted to MSR repeats in mESCs because the cells did not tolerate expression of the RNase enzymes. In addition, GO analysis comparing the MSR-dCas9-Control (-dox) vs MSR-dCas9-Activator (-dox) was not shown, since no cellular pathways were dysregulated. Lastly for Figure 5f, imaging was shown of the induced MEF cells treated with A-485. Images of uninduced A-485 MEF cells was omitted since the percentage of DAPI-dense regions was not altered, however, they were quantified in the stacked bar graph (right panel). Otherwise, all datasets presented in this study did not have omitted data. |
| Replication | For RNA-seq analysis, PCA was performed on the three biological replicates to determine reproducibility. For the other assays, reproducibility was observed between the biological replicates. |
| Randomization | Randomization was not relevant to the study. |
| Blinding | Single blinded experiments were performed for the image analysis of undispersed and dispersed DAPI-dense regions for the doxycycline titration experiment (figure 4c) and the doxycycline induction timecourse experiment (supplementary figure 6c). |

# Reporting for specific materials, systems and methods

We require information from authors about some types of materials, experimental systems and methods used in many studies. Here, indicate whether each material, system or method listed is relevant to your study. If you are not sure if a list item applies to your research, read the appropriate section before selecting a response.

## Materials & experimental systems

| n/a | Involved in the study |
|---|---|
| [ ] | [x] Antibodies |
| [ ] | [x] Eukaryotic cell lines |
| [x] | [ ] Palaeontology and archaeology |
| [x] | [ ] Animals and other organisms |
| [x] | [ ] Clinical data |
| [x] | [ ] Dual use research of concern |
| [x] | [ ] Plants |

## Methods

| n/a | Involved in the study |
|---|---|
| [x] | [ ] ChIP-seq |
| [ ] | [x] Flow cytometry |
| [x] | [ ] MRI-based neuroimaging |

# Antibodies

Antibodies used

All antibodies used were described in the Methods and Supplemental Methods section of the manuscript.

Western blot analysis: Cas9 (Cell Signaling, 14697, 1:1000), GAPDH (Santa Cruz, sc-32233, 1:2000), H3S10ph (Millipore, 06-570, 1:500), H3 (Abcam, ab1791, 1:50000), H3K4me3 (Abcam, ab8580, 1:5000), H3K9me3 (Abcam, ab8898, 1:20000), H3K27me3 (Diagenode, C15410195, 1:2000), H3K36me3 (Abcam, ab9050, 1:2000), H3 pan-acetyl (Abcam, ab47915, 1:5000), GFP (600-141-215, Rockland, 1:1000)

Immmunofluorescence microscopy: H3K4me3 (Abcam, ab8580, 1:2000), H3K9me3 (Abcam, ab8898, 1:2000), H3K27me3 (Diagenode, C15410195, 1:2000), H3K36me3 (Abcam, ab9050, 1:2000), H3 pan-acetyl (Abcam, ab47915, 1:2000), Cas9 (Cell Signaling, 14697, 1:800), HP1alpha (Abcam, ab203432, 1:500), HP1beta(Cell Signaling, 8676, 1:800), HP1gamma(Cell Signaling, 26193, 1:100), RNA Pol II Ser5phos (Cell Signaling, 13523, 1:2000), CREST (Immunovision, HCT-0100, 1:500), HMGA1 (Abcam, ab129153, 1:2000), H3S10ph (Millipore, 06-570, 1:500), GFP (600-141-215, Rockland, 1:1000), mCherry (M11217, Invitrogen, 1:1000)

RNA:DNA hybrid dot blot or IP: S9.6 antibody (Kerafast, Ab01137-23.0, 1:10000)

RNA:DNA IP: S9.6 antibody (Sigma-Aldrich, MABE1095, 2 ug)

Flow cytometry: Ki-67 APC-eFluor 780 (47-5698-82, Invitrogen, 1:2000), Annexin V-APC (Invitrogen, A35122, 1:500)

Validation

All antibodies are commercial and validation was performed by the respective suppliers. Please see the data sheets associated with the antibodies.

# Eukaryotic cell lines

Policy information about cell lines and Sex and Gender in Research

Cell line source(s)

All MEFs and mESCs were established in the lab. MEFs were established as outlined in Rhea et al., 2000; Nature. mESCs were established as outlined in Lehnertz et al., 2003; Current Biology. All cell lines are male.

Authentication

To authenticate that the inducible cell lines, western blot analysis was performed to determine the correct dCas9 fusion protein was expressed.

Mycoplasma contamination

All cells were tested negative for mycoplasma.

Commonly misidentified lines
(See ICLAC register)

There were no misidentified cell lines in this study.

# Plants

Seed stocks

n/a

Novel plant genotypes

n/a

Authentication

n/a

## Flow Cytometry

### Plots

Confirm that:

- [x] The axis labels state the marker and fluorochrome used (e.g. CD4-FITC).
- [x] The axis scales are clearly visible. Include numbers along axes only for bottom left plot of group (a 'group' is an analysis of identical markers).
- [x] All plots are contour plots with outliers or pseudocolor plots.
- [x] A numerical value for number of cells or percentage (with statistics) is provided.

### Methodology

| | |
|---|---|
| Sample preparation | Samples were prepared as described in the methods section "Flow cytometry analysis for cell viability and cell cycle".<br><br>For the cell viability assay, samples were harvested and washed in annexin-binding binding buffer (10 mM HEPES pH 7.4, 140 mM NaCl, 2.5 mM CaCl2). Cells were centrifuged, supernatant discarded, and resuspended in annexin-binding buffer. The cell suspension was then incubated with Annexin V APC and SYTOX Orange for 15 mins at 37C. After incubation, 400 ul of annexin-binding buffer was added and the samples were kept on ice. Prior flow cytometry, samples were filtered through a 35 um mesh strainer.<br><br>For cell cycle analysis, cells were harvested, washed with PBS, and pelleted. The cell pellet was fixed with 1% paraformaldehyde in PBS for 10 minutes at room temperature and then neutralized with glycine (final concentration of 125 mM). Cells were washed with FACS buffer (PBS, 0.5% BSA, 5 mM EDTA). Cells were pelleted and resuspended in permeabilization buffer (00-8333-56, Invitrogen) containing Ki-67 APC-eFluor780 antibody (1:2000) and DAPI (1:1000) and incubated for 30 min on ice. Cells were washed with permeabilization buffer, and resuspended in FACS buffer. Cell suspensions were then filtered through a 35 um mesh prior to flow cytometry |
| Instrument | BD FACSymphony A5 |
| Software | Data was collected using BD FACSDiva and analyzed with FlowJo. |
| Cell population abundance | For both the cell viability and cell cycle study, 100 000 cells were analyzed per condition. |
| Gating strategy | For the cell viability experiment in Supplemental Figure 2a, cells (dead and viable) were initially gated using the SSC-A and FSC-A channels. Single cells were then gated using FSC-H and FSC-A channels. Lastly, the final gate was divided into quadrants showing the percentage of apoptotic/dead cells vs live cells based on the intensity of SYTOX Orange and APC Annexin V staining.<br><br>For the cell cycle study in Supplemental Figure 2b, cells to be analyzed were first gated using the SSC-A and FSC-A channels to exclude cell debris. This population was then further gated using FSC-H and FSC-A channels to obtain single cells. The single cell population was further refined based on DAPI staining. Lastly, the G1, S-phase, and G2/M populations were gated based on DAPI intensity. The M phase population was determined by intensity of Ki-67 labeling. |

- [x] Tick this box to confirm that a figure exemplifying the gating strategy is provided in the Supplementary Information.
